# The Role of Food Parenting Skills and the Home Food Environment in Children’s Weight Gain and Obesity

**DOI:** 10.1007/s13679-015-0139-x

**Published:** 2015-01-28

**Authors:** S. M. P. L. Gerards, S. P. J. Kremers

**Affiliations:** Department of Health Promotion, NUTRIM School for Nutrition and Translational Research in Metabolism, Maastricht University, PO Box 616, 6200 MD Maastricht, The Netherlands

**Keywords:** Food parenting practices, General parenting styles, Intervention studies, Children, Parents

## Abstract

This paper presents an overview to provide readers with an update on the literature about the relation between parental influences (general parenting and food parenting practices) and children’s weight-related outcomes. It first summarizes the evidence regarding the role of food parenting practices in shaping and maintaining children’s nutritional and weight status. It then describes empirical evidence on the relation between general parenting and children’s weight status. This evidence is less convincing, possibly because general parenting has a different, more distal role in influencing child behavior than parenting practices. General parenting may moderate the impact of food parenting practices on children’s nutrition behaviors. Finally, we discuss studies on interventions targeting childhood overweight and obesity. There is no consensus on the optimal intervention targets (i.e., general parenting and/or food parenting practices). Based on the overview, we offer suggestions for future research.

## Introduction

Childhood overweight and obesity are increasing worldwide [1]. Overweight and obese children are at increased risk of developing chronic diseases such as cancer and cardiovascular diseases [2] and are more likely to be obese as adults, compared to those with a healthy weight [3]. Research into determinants of childhood obesity has evolved from a focus on individual level determinants to environmental level determinants [4]. Children are exposed to different environments, for example the home, school and community settings. In younger children in particular, the home setting, and thus their parents, can be considered crucial in determining the children’s weight status [5]. Parents are gatekeepers, for example by determining which food is available at home. Many of children’s nutritional habits are formed in this setting. Although research in this field has been very extensive, research gaps continue to exist.

Parent-related determinants can be classified into two categories: 1. general parenting and 2. specific parenting practices related to particular behaviors, for example food behaviors. General parenting reflects the emotional climate provided by the parents, whereas parenting practices are parental behaviors in a specific context. Parenting practices refer to what parents do, whereas general parenting styles refer to the way they do it [6•]. Furthermore, general parenting styles can be conceived as more distal, higher-order constructs, whereas parenting practices are more proximal determinants of child behavior (see Jansen [6•] and Power [7•]). It should be noted that different authors use slightly different definitions, although with some overall similarities. Both concepts are considered crucial to the development of childhood obesity. The aim of the current narrative review is to provide the reader with an update on the literature about food parenting skills in relation to children’s diet (at ages up to 12 years) and subsequent weight status.

## Food Parenting Practices

Parenting practices are context-specific behaviors of parents, for example relating to food. Food parenting practices are thus parental behaviors intended to influence children’s food intake. A further distinction can be made between parenting practices and feeding styles. Feeding styles refer to ways in which a parent interacts with their child concerning feeding, whereas parenting practices are situation-specific behaviors or strategies to manage how much, when and what children eat, for each type of eating behavior [6•]. Since both concepts are specific parental behaviors related to food, we have summarized both concepts in one section.

Many systematic literature reviews summarizing correlates of children’s diet have concluded that parental factors are crucial in explaining children’s nutrition behaviors [8–12]. Although findings differ between reviews, due to differences in the target group (e.g., different age groups) and differences in the outcome measures (e.g., fruit and vegetables, breakfast, and soft drink consumption), some consistent findings have been reported. Consistent evidence has been found for the relationship between *parental intake* and children’s fruit and vegetable intake [9–11], fat intake [10], breakfast consumption [8], and (although less consistently) soft drink consumption [8–10]. Also, *parental modeling* has consistently been found to be associated with fruit and vegetable intake [9, 10, 12] and soft drink intake [8]. In addition, *home availability and accessibility* have been found to be positively associated with children’s fruit and vegetable consumption [9–11], and children’s soft drink consumption [8], although the empirical evidence for preschoolers is less convincing [12]. *Family rules* were positively associated with children’s fruit and vegetable consumption [9] and *parental limits* were associated with soft drink consumption [8]. A positive association has also been demonstrated between *controlling*/*restrictive practices* and fat intake [10], although no association was found in preschoolers for *restriction* of eating and fruit and vegetable consumption [12]. Inconsistent findings have been reported for the association between *pressuring a child* to eat and their fruit and vegetable intake [12]. *Parental encouragement* was positively associated with children’s fruit and vegetable consumption [9, 10] and, according to one review, negatively associated with energy intake [10]. *Parental permissiveness* was related to greater soft drink consumption [8], less breakfast consumption [8], and less fruit and vegetable consumption [13].

The literature review by Faith and colleagues [14] was one of the first in this field to investigate the association between parental feeding styles and children’s eating and weight status. They found the strongest effects for *feeding restriction* (the extent to which parents restrict their child’s access to foods; as opposed to general feeding control or another feeding domain) and children’s eating and weight status. These findings were confirmed by Clark and colleagues [15], who found that *restriction* was consistently associated with children’s diet and weight. They also found evidence for a causal relationship between parental restriction and childhood overweight. Similarly, Ventura and Birch [16] found substantial evidence for a relation between *restriction* and child weight (positive relation). *Pressure*, *modeling,* and *availability* also seem associated to child weight although the evidence is weak and inconsistent. Wardle and Carnell [17] found that results on the influence of parent’s feeding styles and weight have been conflicting, but that overall, greater *parental control* leads to lower adiposity in the long term or has minimal impact on children’s weight.

Hurley and colleagues [18] made a distinction between *responsive feeding* (i.e., parental guidance with recognition of the child’s cues of hunger and satiety) and *non-responsive feeding* (i.e., a lack of reciprocity between the parent and the child). Non-responsive feeding can be further subdivided into parents taking excessive control toward the feeding situation (pressuring or restricting food intake) or children controlling the feeding situation (indulgent feeding). The most frequent finding was an association between parental feeding control and children’s weight status. Studies have also suggested a positive relationship between indulgence and children’s weight status.

We found one systematic literature review investigating the role of fathers’ child feeding practices [19]. Although the authors concluded that the evidence on the role of fathers is scarce, they found that fathers do consider themselves responsible for feeding their children and helping to prepare meals. Moreover, they found that fathers focused more on getting children to eat and were less concerned about the specific foods consumed than mothers. Compared to mothers, fathers were more likely to pressure children to eat or restrict food for weight-related reasons, and were less likely to place limits on snacks or to ensure the consumption of a variety of foods and daily access to fruits and vegetables, reflecting an unresponsive feeding pattern. As regards children’s weight, fathers reported using higher levels of *restriction* for children with a higher BMI [19].

The majority of the systematic reviews described above have recognized a gap regarding the measurement of food parenting practices. Vaughn and colleagues [20•] performed a systematic review on measures related to practices and assessing the quality of these practices. They found 71 unique instruments to measure food parenting practices, measuring a variety of constructs and representing different levels of quality. They also tried to make a start with conceptualizing the relevant constructs.

In addition to these systematic literature reviews, there have been other initiatives to structure the complex range of food parenting practices. For example, Baranowski and colleagues [21] tried to assess the dimensional structure of parenting practices related to children’s vegetable intake, in order to better understand the co-occurrence of food parenting practices. Acceptable fit was obtained with three factors (responsiveness, control, and structure), but only when effective and ineffective practices were analyzed separately, suggesting separate dimensions for effective and ineffective practices.

Gevers et al. [22•] performed a Delphi study to clarify food parenting practice concepts related to snacking, in order to reach consensus on the concepts of relevant practices. The reason for this study was the lack of clarity about the full range of food parenting practices and what is meant by the different practices. An extensive set of practices and corresponding prototypic parenting practices was identified (see Table 1). However, they also found that there is still a lack of clarity about what is meant by the different food parenting practices that have been reported. Although the evidence on some of these practices is quite convincing, the influence of others is not as straightforward. The authors state that the next step might be to conceptualize the identified food parenting practices, in order to fully understand food parenting practices.

**Table 1:** List of food parenting practices based on Gevers et al. [22•]

Concept	Prototypic description of parenting practice
Accessibility	Storing food in a location the child cannot access on his or her own
Availability	Having healthy foods at home
Discussing	Discussing the availability of food with the child
Educating	Teaching the child about foods
Emotional feeding	Using food in response to the child's emotional distress
Encouragement	Encouraging the child to eat a large variety of foods
Instrumental feeding	Using foods to persuade the child to do something
Involving	Allowing the child to assist in preparing food
Meal routines	Eating meals together as a family
Modeling	Eating healthy foods in the presence of the child
Monitoring	Keeping track of the food the child eats
Permissiveness	Giving in to the child's opposition to eat healthy food
Pressure to eat	Pressuring the child to eat healthy foods
Providing feedback	Providing a positive or negative response on the food a child has consumed
Rewarding	Offering the child toys or other non-food rewards for healthy eating
Rules	Setting explicit rules, for example about what kind and how much food the child is allowed to eat
Structure	Giving the child food at fixed times
Visibility	Having healthy foods where they can easily be seen

Some studies have focused on determinants of food parenting practices. A recent systematic review by McPhie and colleagues [23] summarized predictors of maternal feeding practices. The determinants they found were maternal parenting (parenting control and demandingness (i.e., the extent to which parents control their children)), maternal personal characteristics (SES, ethnicity), and maternal general and eating-related psychopathology. The Model of Goal Directed Vegetable Parenting Practices has been used to understand parenting practices related to vegetable consumption [24]. This model was based on the Model of Goal Directed Behavior (which added anticipated emotions and desire to the Theory of Planned Behavior) and Self Determination Theory. The study found that 40.5 % of the variance was explained by the model using the ineffective parenting practices [25] and 48.6 % by the model of effective parenting practices [26]. Habits showed the strongest relationships with the ineffective and effective parenting practices related to vegetable consumption.

In addition to research investigating specific parental behaviors, another approach is to structure the reciprocal influence of family members, recognizing that family members are part of a larger system. The family health climate, a relatively new concept in the literature, is defined as the shared perceptions and cognitions concerning health and health behavior [27]. This variable represents an attribute of the family as a whole, i.e., a family-level variable, which affects the health behavior of family members. This family health climate may thus determine the environment in which parenting practices are formed.

## General Parenting

General parenting (or parenting style) concerns parenting across situations and reflects the emotional climate in which children are raised [28]. It determines the behavioral expression between parent and child, and is a reflection of parents’ attitudes, beliefs, and behaviors. General parenting was traditionally characterized by the degree to which parents are responsive and demanding toward their children [29]. Responsiveness is the extent to which parents respond to their child’s needs, also called warmth, involvement, or nurturance. Demandingness is the extent to which parents control their children, also called behavioral control or restrictiveness. Cross-tabulating these two dimensions allow four different parenting styles to be distinguished: authoritative (parents who are both demanding and responsive), authoritarian (parents who are demanding but low in responsiveness), indulgent (parents who are responsive but not demanding), and neglectful (parents who are neither responsive nor demanding). Authoritative parents are characterized by expressing warmth and emotional support, but also use clear, open communication [29]. In addition to this categorization, Skinner and colleagues [30] identified three core dimensions of parenting: parental warmth vs rejection, parental structure vs chaos, and autonomy support vs coercion, thus identifying a third dimension of parenting. Sleddens et al. [31•] elaborated on these dimensions in their development of a comprehensive parenting model, i.e., parental nurturance, structure, and control. They further distinguished different forms of control: behavioral control (controlling practices supporting children’s development), overprotection (excessive parental involvement), and coercive control (parental dominance). These five parenting constructs describe major differences in parental behavior and they are further subdivided into sub-constructs.

Ventura and Birch [16] were the first to summarize evidence regarding parenting and children’s diet and weight status. They found an association in two of the four studies between general parenting and children’s weight status. They also concluded, however, that it is important to recognize that general parenting is responsive to and influenced by child characteristics. The majority of the studies investigating the association between general parenting and children’s weight were cross-sectional, limiting the possibility to identify causal relationships. Skouteris and colleagues [32] continued to work on the idea that it is important to focus more on bi-directional perspectives on parent-child relationships. So rather than solely focusing on a top-down approach in which parents influence the child, researchers should focus on the parent-child dyad and recognize that children’s development is shaped by the reciprocal nature of both parent-level and child-level factors. In their review, they reported on five studies examining the influence of parent-child interactions as risk factors for childhood obesity.

In a literature review by Sleddens and colleagues [33] investigating the evidence for associations between general parenting and children’s weight-related outcome measures, authoritative parents were consistently found to have healthier children in terms of weight-related outcome measures (nutrition, physical activity, and weight status) than children raised by parents using the other parenting styles. In line with this, Blissett [13] concluded that lack of control, i.e., a permissive parenting, may be associated with unfavorable fruit and vegetable intake by children. Berge et al. [34] also concluded that authoritative parenting is associated with favorable outcomes in terms of child obesity, dietary intake, and physical activity.

Pinquart [35•] conducted a meta-analysis on the association between general parenting and parent-child relationship and children’s weight status. Their study found that a positive parent-child relationship and higher levels of parental responsiveness were associated with lower weight, healthier eating, and more physical activity by the child. Furthermore, parental demandingness, overprotection, psychological control, inconsistency, and parenting styles showed associations with some of the outcome variables. However, most effects were very small, so this study did not support the idea that targeting these concepts in intervention studies might be successful. Although the number of available studies was limited, they assumed that reducing parenting inconsistency may be a better intervention target.

**Fig. 1:**
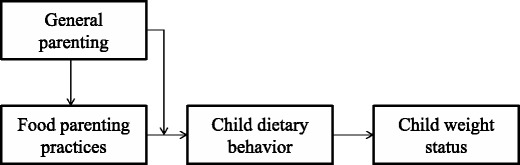
A model of relation between general parenting, food parenting practices, and children’s outcomes

Social ecological theory hypothesizes higher-order moderation processes [36], thus implying that parenting factors at higher, more distal, levels (general parenting) can moderate the impact of factors at a lower level (food parenting practices). As a result, a factor at a higher level forms the context in which proximal parenting processes operate. There is some early evidence regarding the relationship between general parenting and parenting practices. For example, Sleddens et al. [37] investigated the moderating role of parenting and found that the associations of encouragement and covert control with healthy child dietary behaviors were stronger for children who reared in a positive parenting context. Rodenburg et al. [38] found that associations between parental feeding styles and outcome measures depended on the degree of psychological control and behavioral control. Similarly, Tung and Yeh [39] found that parenting styles have a moderating effect on parenting practices and children’s weight status. In their study, monitoring children’s dietary intake seemed to be more effective in terms of weight control among the authoritative mothers than among the authoritarian mothers. These studies provide some evidence that general parenting sets the context in which food parenting practices are performed (see Fig. 1). These findings may explain the inconsistent findings in different studies. Depending on the context, food parenting practices might have a stronger or weaker influence on children’s nutritional behavior and eventual weight status.

## Intervention Studies

Several systematic reviews have summarized evidence regarding family- or home-based interventions to prevent or treat childhood obesity. Knowlden & Sharma [40] performed a systematic review in 2012 on family- and home-based interventions targeting childhood overweight and obesity. They found nine studies, eight of which reported significant intervention effects. They concluded that more interventions should be developed targeting parents as the primary agent of change. In contrast, a recent review by Showell and colleagues [41] on home-based intervention studies showed that the strength of the evidence is low, i.e., only a small number of studies (n = 6) examined childhood obesity prevention programs in the home setting and none of these studies showed significant intervention effects on weight-related outcomes. Three studies reported effects on diet or physical activity outcomes. Skouteris et al. [42] conducted a systematic review about parental variables targeted in intervention studies. They found 11 studies. Parental knowledge about nutrition and role modeling were the most frequently targeted intervention components. Teaching parents about nutrition and fostering healthy lifestyle behaviors were found to result in improved parental knowledge and parent and child behaviors and/or child BMI.

Some interventions focus particularly on general parenting in addition to parenting practices. A systematic review we performed in 2011 identified only seven intervention studies targeting general parenting in order to prevent or treat childhood obesity [43]. The studies we reviewed all found positive intervention effects on at least one outcome measure related to children’s weight. Since then, more intervention studies have been published addressing general parenting. These studies have shown mixed results. Some found promising effects on children’s weight-related outcome measures [44–49], while one other study found no effect on weight-related outcomes [50]. Van Ryzin and Nowicka [51] found that an indirect effect of a general parenting intervention on obesity in adolescents could be explained by the parent-youth relationship quality. They showed that family processes may play a role in the likelihood of childhood obesity.

An interesting question arising from the distinction between parenting practices and general parenting is whether we should focus on general parenting or specific parenting practices [7]. We have previously argued that targeting general parenting in addition to parenting practices might be necessary [43]. Since general parenting and parenting practices are variables at different levels, the incorporation of general parenting components can help to increase the impact of the intervention elements aimed at practices. However, other authors (e.g., [52•]) disagree and state that future prevention and treatment interventions should aim at specific parenting practices rather than at general parenting, to increase the effectiveness of the interventions.

## Recommendations

With regard to food parenting practices, it is important that consensus is reached on a conceptual model including the most relevant food parenting practices [20•]. For some practices, it remains unclear whether they positively or negatively impact child’s weight. Development and use of a comprehensive instrument to assess the full scope of relevant food parenting practices will help the field in refining such conceptual models and empirical investigations.

We think that the general parenting field has made some progress regarding conceptualization. Three important domains are widely recognized, and as a result a conceptual model and corresponding measurement tools have been developed (e.g., [31•]). However, it is important to conduct more research into the validity of such tools across a range of target groups in terms of age, gender, and ethnic and cultural background.

It is important to include both food parenting practices and general parenting, in order to improve our understanding of the contextual or higher-order processes [53•]. We recommend higher-order moderation approaches as having significant value for understanding the complex process of parent-child interactions, in relation to the development of childhood overweight [53•]. Note that such studies need large sample sizes in order to test for (higher order) interactions and execute subsequent stratified analyses.

More research should be directed toward optimizing parental involvement in intervention studies. This is an important first step for intervention research. To date, most interventions with a parental component have failed to involve substantial amounts of parents throughout the recruitment and intervention implementation process. Intervention studies should thus be designed to closely fit parental needs, in addition to those of the child. For this purpose, both child and parental views should be investigated as important starting points of intervention development.

Family-based interventions should be developed in such a way that positive parenting practices are promoted, while simultaneously addressing general parenting. Hereby, parents learn which practices might be advantageous while realizing the optimal context for the implementation of such practices. However, to date, it remains unclear which methods and strategies are most effective in providing parents with useful tools to adopt and implement optimal positive parenting practices.

It is important that intervention studies recognize parental differences in needs regarding the intensity of parenting support. Some parents only need confirmation of their current parenting behaviors, while others need intensive support in how to healthily raise their child. The appropriate level of intervention may also be dependent on the child’s weight status and temperament. We would therefore advocate for a system of interventions, in which interventions are in place at all levels of family needs (see, e.g., [54]). In such a systems approach, it is important that different settings in which the child lives (home, school, community) collaborate in influencing the child in a healthy way (i.e., integral approach [55]). The optimal delivery mode should fit parental needs. Some may prefer educational sessions, in the form of group sessions. Greater intervention intensity may be necessary to achieve beneficial and long-term effects on weight [41], while the added value of home visits and online interventions should be further investigated.

## Conclusions

A rapidly increasing amount of research is devoted to the study of the relation between parental behaviors and children’s weight-related outcomes. We advocate a holistic conceptual view which addresses the broad scope of food parenting practices, a shift in research focus toward the investigation of the role of contextual higher-order mechanisms, and intervention studies addressing optimal integral collaboration, as well as intervention intensity, delivery mode, and contents tailored for distinct target groups.
